# Ferrocene-functionalized polyheteroacenes for the use as cathode active material in rechargeable batteries†

**DOI:** 10.1039/c8ra00129d

**Published:** 2018-04-17

**Authors:** Pierre-Olivier Schwartz, Sebastian Förtsch, Elena Mena-Osteritz, Dagmar Weirather-Köstner, Mario Wachtler, Peter Bäuerle

**Affiliations:** Institute of Organic Chemistry II and Advanced Materials, Ulm University Albert-Einstein-Allee 11 89081 Ulm Germany pierreolivier.schwartz@gmail.com peter.baeuerle@uni-ulm.de; ZSW – Zentrum für Sonnenenergie-und Wasserstoff-Forschung Baden-Württemberg Helmholtzstr. 8 89081 Ulm Germany +49-731-502-2840

## Abstract

Herein we report the synthesis and characterization of new conjugated polymers bearing redox-active pendant groups for applications as cathode active materials in secondary batteries. The polymers comprise a ferrocene moiety immobilized at a poly(cyclopenta[2,1-*b*:3,4-*b*′]dithiophene) (pCPDT, P1) or a poly(dithieno[3,2-*b*:2′,3′-*d*]pyrrole) (pDTP, P2) backbone *via* an ester or an amide linker. Electrochemical and oxidative chemical polymerizations were performed in order to investigate the redox behaviour of the obtained polymers P1 and P2 and to synthesize materials on gram-scale for battery tests, respectively. During galvanostatic cycling in a typical battery environment, both polymers showed high reversible capacities of 90% and 87% of their theoretical capacity and excellent capacity retentions of 84% and 97% over 50 cycles.

## Abstract 2

Redox-active ferrocene moieties were immobilized on conjugated polyheteroacenes for the application as cathode active material in organic secondary batteries.

## Graphical abstract

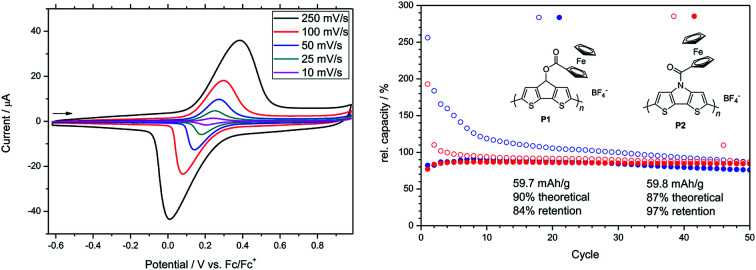

## Introduction

In the last decade, a great deal of interest has been devoted to the synthesis and investigation of new conjugated oligomers and polymers due to their application in various fields, such as light-emitting diodes (OLEDs),^1^ plastic lasers,^2^ field-effect transistors,^3^ photovoltaic devices,^4^ or as active materials in organic secondary batteries.^5^ Among the emerging classes of electroactive conjugated materials, fused heteroacenes, such as 4*H*-cyclopenta[2,1-*b*:3,4-*b*′]dithiophene (CPDT) 1 and 4*H*-dithieno[3,2-*b*:2′,3′-*d*]pyrrole (DTP) 2 have attracted considerable attention in numerous applications.^6^ Both building blocks consist of a bithiophene unit, which is bridged at the 3,3′-position by a methylene or amino group. Because of the fully coplanar structure, the intrinsic properties based on bithiophene can be altered leading to more extended conjugation in the ground state and more rigid structures. Hence, the corresponding polymers pCPDT and pDTP exhibit an increased electrical conductivity, a reduced band gap, and closer intermolecular interactions. Furthermore, the introduction of solubilizing aliphatic side chains^7^ or other types of functional groups at the bridging atom^8^ allows for the functionalization of the conjugated unit without affecting their coplanarity. As potential new application pCPDTs and pDTPs are good candidates for cathode active materials in organic batteries. Indeed, they possess many desirable characteristics for this type of application: they can be reversibly doped, can be prepared in thin-film form, and they possess a good conductivity. However, they often exhibit low doping levels resulting in small redox capacities and slow kinetics at the electrode. To overcome this limitation, an alternative approach is the use of redox active species attached as pendant groups to the corresponding conjugated polymer.

Bis(η^5^-cyclopentadienyl)iron(ii) (ferrocene, Fc) is a prominent sandwich-like organometallic compound, which is widely used as standard in electrochemical analyses because of its excellent stability and super-fast electrochemical response, among others.^9^ The oxidation to the ferricenium cation [Fe(C_5_H_5_)_2_^+^, Fc^+^] was proven to be fully reversible and the one-electron exchange rate constant has been determined to be 7 × 10^−1^ cm s^−1^.^10^ Initially reported by Masuda *et al.* in 2008,^11^ the possible use of ferrocene-containing polymers as cathode material in organic batteries has been investigated by several groups, since then.^12^ However, even if the tested materials showed promising properties (quick recharge, high power density, stable voltage plateau), most of them suffered from capacity fading due to their low molecular weights. In light of these considerations, we aimed to synthesize conductive polymers from thiophene-based heteroacenes comprising pendant ferrocene substituents. This strategy should allow for reducing the solubility of the active material (decreased solubility due to increasing molecular weight), while maintaining a good electric connection of ferrocene to the current collector in the electrode (due to the electronic conductivity of the polymer backbone).^13^

The charge/discharge mechanism of ferrocene-functionalised materials is based on the reversible oxidation of ferrocene (Fe^2+^) to ferricenium (Fe^3+^). To maintain charge neutrality in the electrode, an anion from the electrolyte is “inserted” by the electrode. Also the conductive polymer backbone, which can be reversibly oxidised/reduced gives some contribution to charge storage (again *via* anion “insertion”). In a full cell the ferrocene-functionalised cathode can be combined with a Li insertion/conversion or a Li metal anode. The full cell thus constitutes a dual-ion system with different types of ions responsible for the charge storage in anode and cathode. Other dual-ion systems described in the literature are for instance organic radical batteries^14^ or dual carbon batteries.^15^

In this publication, we report synthesis and characterization of two new polymers P1 and P2, which combine pCPDT or pDTP as conjugated polymer backbone and ferrocene moieties as redox-active pendant group. The polymerization of the two corresponding monomers by means of electrochemical and oxidative chemical polymerization is described. First investigations on both polymers as active material in organic batteries are also reported.

## Results and discussion

### Monomer and polymer synthesis

**Scheme 1:**
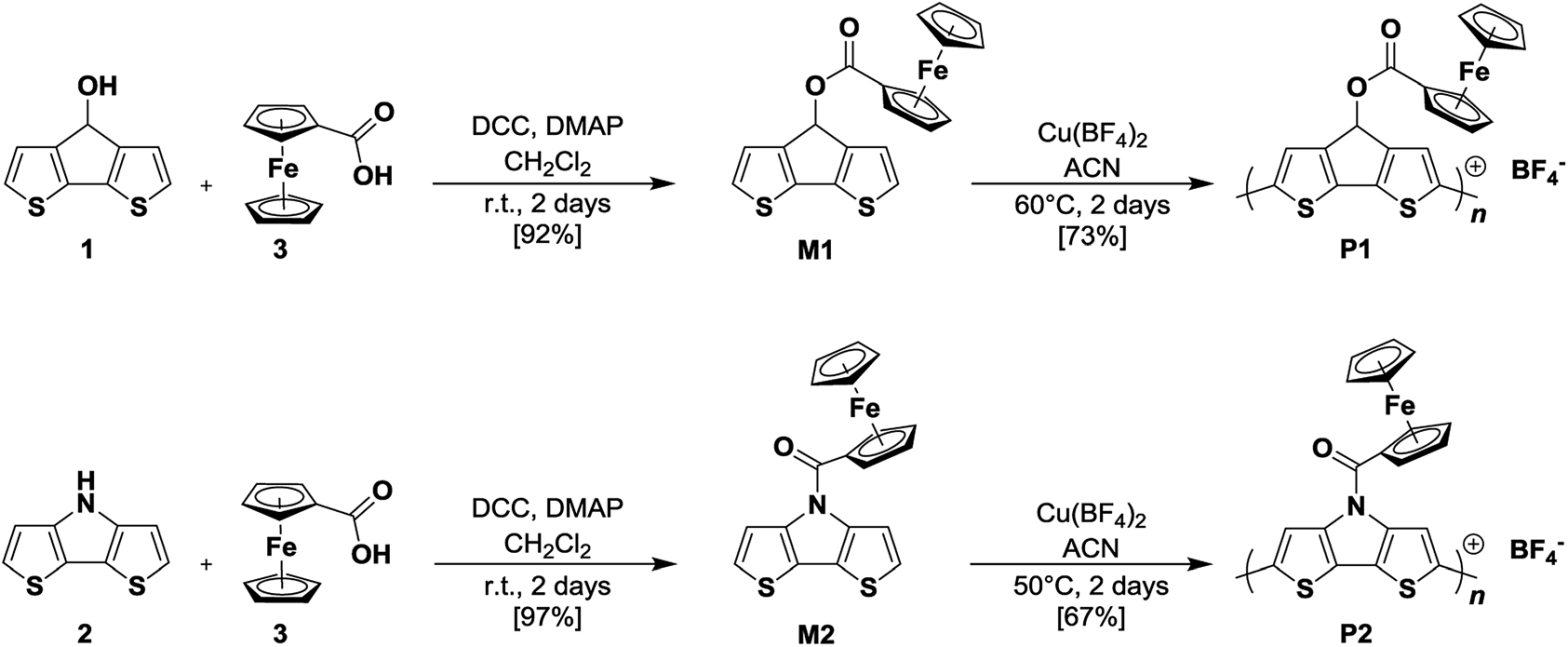
Syntheses of CPDT–Fc M1, DTP–Fc M2, and corresponding polymers pCPDT–Fc P1 and pDTP–Fc P2.

The synthetic routes and the chemical structures of monomers CPDT–Fc (M1) and DTP–Fc (M2), as well as the corresponding polymers pCPDT–Fc (P1) and pDTP–Fc (P2) are shown in Scheme 1 (see for more details in ESI†).

Monomer M1 was synthesized in a Steglich esterification reaction^16^ of 4*H*-cyclopenta[2,1-*b*:3,4-*b*′]dithiophen-4-ol 1 with commercially available ferrocene carboxylic acid 3 in the presence of the dehydrating agent dicyclohexylcarbodiimide (DCC) and 4-dimethylaminopyridine (DMAP). Using this route, M1 was obtained in a yield of 92% when the reaction was run at room temperature for two days. The same strategy was applied for the synthesis of M2 while replacing 1 by 4*H*-dithieno[3,2-*b*:2′,3′-*d*]pyrrole 2. Under the same conditions, M2 was obtained in a yield of 97% after purification. Both monomers, CPDT–Fc M1 and DTP–Fc M2, were fully characterized by means of NMR spectroscopy, elemental analysis, and mass spectrometry.

**Fig. 1:**
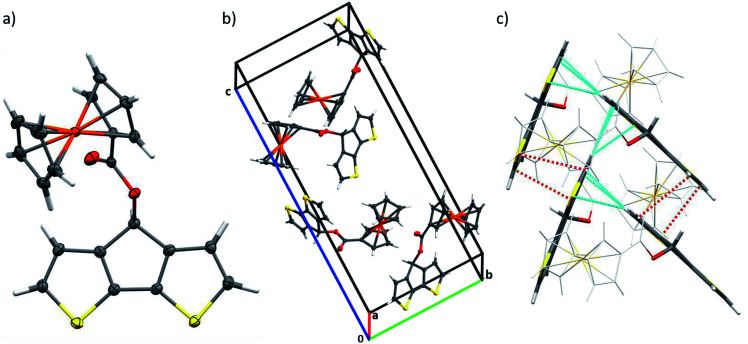
Crystal structure of CPDT–Fc M1: (a) molecular structure, (b) unit cell, and (c) intermolecular contacts: π–π interactions (red dotted lines) and H–π herringbone interactions (cyan dotted lines). Displacement ellipsoids are drawn in (a) and (b) at the 50% probability level. The ferrocene units in (c) are shown in light grey for clarity.

We were able to grow single crystals from monomer M1 and performed X-ray single crystal structure analysis (Fig. 1, Table S1†). Ferrocene-functionalized cyclopentadithiophene M1 crystallizes in the *P*2_1_2_1_2_1_ symmetry group with four molecules in the unit cell and is ordered along the three main directions by two-fold screw axes symmetry operation (Fig. 1b). As expected the CPDT–π-system reveals coplanarity and forms a 61.2° angle with the ferrocene moiety symmetry axis. The bond distances in the aromatic CPDT unit are in good agreement with the ones published for related unsubstituted CPDT moieties (Table S2†).^17^ On the contrary, the bond distances and angles for the ferrocene unit are in good agreement or only slightly larger than the published data for related benzyl-1-ferrocenoate.^18^ In the unit cell, two relevant stacking patterns of the molecules with intermolecular distances below the van der Waals radii can be observed (Fig. 1c, Table S3†): π–π stacking between partially overlapping CPDT aromatic parts (3.349–3.486 Å) and a herringbone arrangement of aromatic moieties with strong H–π interactions (2.804–2.98 Å). Each molecule interacts therewith with up to six neighboring molecules.

Oxidative chemical polymerization of monomers M1 and M2 was performed in order to synthesize corresponding polymers pCPDT–Fc P1 and pDTP–Fc P2 on gram-scale (Scheme 1). For both monomers, the polymerization process was conducted in acetonitrile (ACN) with copper(ii) tetrafluoroborate as oxidant.^19^ After two days, the oxidized polymer chains complexed with BF_4_^−^ counter anions were obtained as precipitates. Polymers P1 and P2 were recovered *via* centrifugation and purified by extensive washings with several different solvents. The pure products were obtained as a black powder for pCPDT–Fc P1 and as a dark blue powder for pDTP–Fc P2 in yields of 73% and 67%, respectively. Due to the insolubility of the polymers in any common organic solvent, they could only be characterized in the solid state. By using FT-IR spectroscopy, it was possible to confirm the characteristic chemical structures of both polymers after the oxidative chemical polymerization (ESI, Fig. S2†). Thus, the stretching bands of the ester moiety (C=O at 1715 and C–O at 1264 cm^−1^) in P1 and the amide group (C=O at 1637 cm^−1^) in P2 as well as band of ferrocene (Fc-Cp at 499 and 488 cm^−1^) were clearly present. The thermal stability was investigated by means of thermogravimetric analysis (TGA) and differential scanning calorimetry (DSC). The TGA results, which correspond to the evaluation of the weight loss over time while the sample is heated, indicated a good stability of both polymers up to 270 °C (less than 5% mass loss, ESI, Fig. S3†). At higher temperatures, the polymers started to slightly degrade and at the end of the measurement (800 °C) an amount between 60 to 70% of an inert residue still remained. In DSC analysis, both derivatives were heated to 300 °C (ESI, Fig. S4†). A similar tendency was observed with no significant endothermic transition peak within the cycles. As a consequence, we concluded that both polymers, pCPDT–Fc P1 and pDTP–Fc P2, were very stable and did not display major degradation up to 270 °C.

### Electropolymerization

The redox behaviour of both monomers was investigated electrochemically by means of cyclic voltammetry (CV). The measurements were carried out in 10^−3^ M solutions of each monomer M1 or M2 with tetrabutylammonium hexafluorophosphate (TBAPF_6_) dissolved in dry acetonitrile (0.1 M) as electrolyte (ESI, Fig. S5†). The CVs revealed a first electrochemically reversible redox wave at 0.25 V *vs.* Fc^+^/Fc [(*E*^a^_p_ + *E*^c^_p_)/2] for M1 and at 0.18 V for M2. This redox process was attributed to the oxidation of the ferrocene unit to the ferricenium cation and the corresponding reversible reduction. A second non-reversible oxidation signal with peak potentials at 0.81 V and 0.84 V *vs.* Fc^+^/Fc for M1 and M2 was observed, respectively. These processes were due to the oxidation of the polymerizable backbone units and were in accordance with previous reports on alkyl-substituted CPDTs and DTPs.^20^

**Fig. 2:**
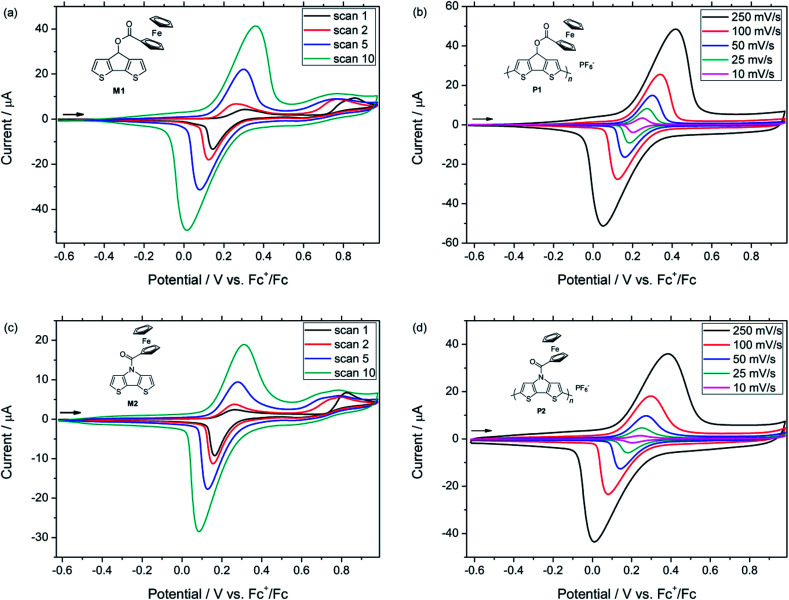
Electropolymerization of M1 (a) at a scan rate of 200 mV s^−1^ and M2 (c) at a scan rate of 100 mV s^−1^ in 0.1 M TBAPF_6_ in CH_2_Cl_2_ (monomer concentration 10^−3^ M). Cyclic voltammograms of pCPDT–Fc P1 (b) and pDTP–Fc P2 (d) in 0.1 M TBAPF_6_ in CH_2_Cl_2_ applying different scan rates.

It is noteworthy that no polymerization of the monomers was observed after several scans in acetonitrile. In order to follow the formation of their polymer films, monomers M1 and M2 had to be subjected to potentiodynamic electrochemical polymerization in a solution of TBAPF_6_ in dichloromethane as the solvent (Fig. 2). For M1, the polymerization started at 0.72 V (*vs.* Fc^+^/Fc) with the oxidation of the CPDT moiety, while the ferrocene group of the monomer was reversibly oxidized and reduced at 0.31 and 0.14 V, respectively. By increasing the number of cycles, the gradual formation of the polymer film could be monitored, while the monomer was successively consumed. Indeed, the potentials of the oxidation peak maxima varied from the first to the second cycle, in the case of CPDT from 0.86 to 0.77 V and from 0.31 to 0.26 V for the ferrocene moiety. This behaviour is a common feature in conducting polymers since the oxidation of the polymer film requires a lower potential than the oxidation of the monomer. It is due to the increase of the HOMO energy level of the polymer (in comparison with the corresponding monomer), which occurs during the polymerization process. After 10 scans, polymer pCPDT–Fc P1 was strongly adhered to the working electrode and provided a homogeneous golden to brownish film (observed by optical microscopy). For M2, a similar trend was observed with the polymerization starting at 0.73 V (*vs.* Fc^+^/Fc) due to the oxidation of the DTP moiety, while the ferrocene group of the monomer was oxidized and reduced at 0.26 and 0.16 V, respectively. Again, it was possible to follow the formation of the polymer film through the first scans and after 10 scans a dark blue film of pDTP–Fc P2 was strongly adhered to the electrode.

In order to electrochemically characterize the deposited polymers P1 and P2, a monomer-free electrolyte solution was used (0.1 M TBAPF_6_ in CH_2_Cl_2_) and the measurements were performed at different scan rates (Fig. 2, ESI, Fig. S6 and S7†). The cyclic voltammograms of the films showed a different behaviour depending on the scan rates. First of all, the charging of the conjugated polymer, which is concomitant with the transition from the semiconducting to the conducting state of the polymer, started at around −0.24 V *vs.* Fc^+^/Fc for P1 and at −0.21 V for P2. By increasing the scan rate, the anodic peak potentials of the conjugated backbone and the ferrocene moieties were considerably shifted to higher potentials, whereas the reduction of the ferrocene unit was shifted to lower potentials, respectively. This tendency can be attributed to the increased diffusion time of the counter anions. In follow-up measurements, the stability of polymer P1 and P2 was tested. After 30 cycles (scan rate of 100 mV s^−1^), 94% and 90% of the original capacity was still observed for pCPDT–Fc P1 and pDTP–Fc P2, respectively. These results proved the good stability of the electrochemically synthesized polymers.

**Fig. 3:**
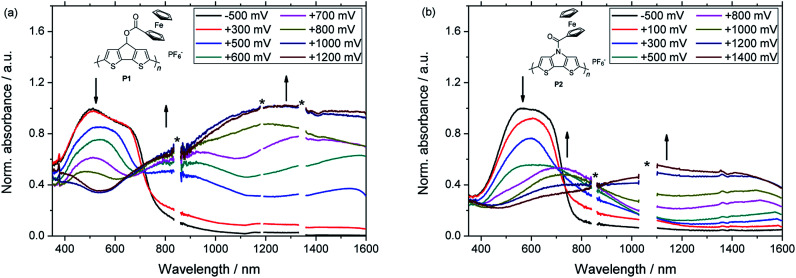
UV-vis-NIR spectra obtained from spectroelectrochemical measurements on polymer P1 (a) and P2 (b). Applied voltages are referenced *vs.* Ag/AgCl. Arrows show the spectral changes upon increasing the applied voltage. Artefacts are marked with * in the spectra.

Subsequently, films of P1 and P2 were investigated by spectroelectrochemistry using previously described equipment (Fig. 3).^21^ Notably, the applied platinum working electrode ensured that the same materials were obtained for the electrochemical and the spectroelectrochemical characterizations, respectively. The partly oxidized films were initially neutralized by applying a negative potential and both polymers showed a broad absorption band in the visible range of the UV-vis-NIR spectrum. Similar energy gaps were determined from the onset absorptions of the polymers (*E*_g_ = 1.59 eV for P1, 1.63 eV for P2) and also the energy levels of the Frontier molecular orbitals were in a similar range (Table 1). During the spectroelectrochemical measurements, the applied potentials were gradually increased firstly leading to the formation of polarons. As a consequence, the absorption band in the visible region decreased and two new bands at higher wavelengths emerged. In their fully oxidized forms, polymers P1 and P2 exhibited only one broad band, which extended over a wide range of the NIR region and indicated the presence of bipolarons.

**Table 1:** Optoelectronic properties of polymers P1 and P2. The values of *λ*_max_ and the energy gap *E*_g_ were obtained from UV-vis-NIR spectra of the neutral polymer films. The maxima *λ*_max,p_ of the polaronic and *λ*_max,bp_ of the bipolaronic species were obtained from spectroelectrochemical measurements. The HOMO energy levels were determined from the onset oxidation potential. The LUMO energy levels were obtained from the corresponding HOMO energy level and the energy gap *E*_g_

Polymer	*λ*_max_ (nm)	*λ*_max,p_ (nm)	*λ*_max,bp_ (nm)	HOMO (eV)	LUMO (eV)	*E*_g_ (eV)
P1	512	828, 1533	1276 (br)	−4.86	−3.27	1.59
P2	570	710, 1511	1115 (br)	−4.89	−3.26	1.63

### Organic battery investigations

**Fig. 4:**
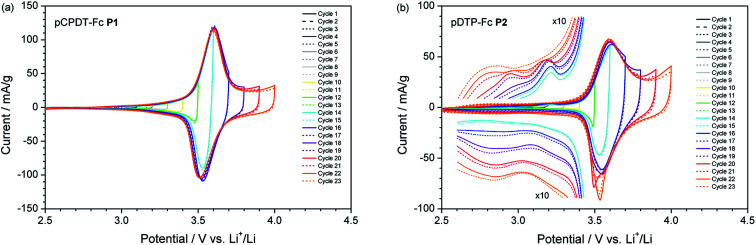
Cyclic voltammograms of (a) pCPDT–Fc P1 and (b) pDTP–Fc P2 in 1 M LiPF_6_/EC : DMC (1 : 1 by weight) at a scan rate of 0.1 mV s^−1^ and with a stepwise increase of the upper vertex potential.

In order to assess the suitability of the novel polymers for organic batteries, the active materials were processed into composite electrodes and tested in a typical Li-ion battery environment with a 1 M solution of LiPF_6_ in ethylene carbonate (EC) : dimethylcarbonate (DMC) (1 : 1 by weight) as electrolyte (for details see ESI†). Fig. 4 presents the cyclic voltammograms obtained at a low scan rate of 0.1 mV s^−1^, which ensures the complete reaction of the electrode. Both polymers, pCPDT–Fc P1 (Fig. 4a) and pDTP–Fc P2 (Fig. 4b), showed a dominating reversible wave at 3.65 and 3.62 V *vs.* Li^+^/Li, respectively. These values are higher than the potential for the Fc^+^/Fc couple dissolved in the same electrolyte (3.24 V *vs.* Li^+^/Li).^22^ The shift to higher potentials can be explained by the electron-withdrawing effect of the ester or amide group, through which the ferrocene units are coupled to the polymer chains.

Apart from the Fc^+^/Fc main peak, some additional weak features were observed. Both polymers showed weak anodic and cathodic base currents at potentials higher than 2.8 to 3.0 V *vs.* Li^+^/Li. These are ascribed to the reversible oxidation of the polymer chain. For polymer pDTP–Fc P2 additional peaks appeared below 3.0 V *vs.* Li^+^/Li in the anodic scan and below 3.1 V *vs.* Li^+^/Li in the cathodic scan, as soon as the upper vertex potential reached 3.8 V *vs.* Li^+^/Li. One possible explanation could be the occurrence of irreversible side reactions or follow-up reactions. With a further increase of the upper vertex potential the two extra peaks grew in intensity and shifted to lower potentials. Concomitantly with the appearance of the onset of the new process above 3.8 V *vs.* Li^+^/Li the coulombic efficiency, obtained by integration of the CV curve, drops below 90%.

**Fig. 5:**
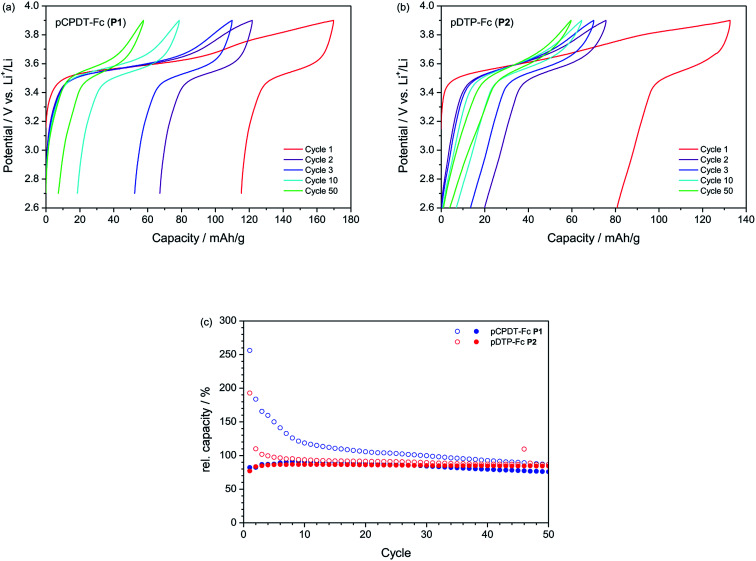
Galvanostatic cycling performance of pCPDT–Fc P1 (a and c) and pDTP–Fc P2 (b and c) in 1 M LiPF_6_/EC : DMC (1 : 1 by wt.), at a current rate of 0.1C. In (c) the open symbols denote the charge capacities and the closed symbols denote the discharge capacities relative to the theoretical capacities.

The results from galvanostatic cycling tests are summarized in Fig. 5. The capacities shown in Fig. 5c are normalised to the theoretical capacity of the materials (66.3 mA h g^−1^ for pCPDT–Fc P1 and 68.8 mA h g^−1^ for pDTP–Fc P2) in order to facilitate a direct comparison (the theoretical capacities are based on a one-electron process of the ferrocene group and do not take into account contributions from the conductive polymer backbone). With a value of 59.7–59.8 mA h g^−1^ both polymers P1 (90%) and P2 (87%) reached reversible capacities (equals discharge capacities), which were very close to their theoretical capacity, respectively. Polymer P1 showed good cycling stability with a capacity retention of 84% (the capacity retention is here defined as reversible capacity in cycle 50 relative to that in cycle 10). Polymer P2 showed an even better cycling stability with a capacity retention of 97%, which ranks among the best performances reported for organic electrode materials, so far (*cf. e.g.*ref. 5*c* with an extensive review of the electrochemical behaviour of more than 70 polymeric materials). P1 and P2 fit thus well to other ferrocene-substituted polymers, such as poly(vinyl ferrocene)^11^ with an initial capacity of 83% of the theoretical capacity and a capacity retention of 95% after 300 cycles, or poly(fluorenylethynylene ferrocene)^12*e*^ with an initial capacity of 95% of the theoretical capacity and a capacity retention of 88% after 100 cycles.

Unfortunately, the materials P1 and P2 suffered from significant irreversible capacities (difference between charge and discharge capacities). The elevated initial irreversible capacity was, furthermore, not restricted to the first cycle, as for many inorganic electrode materials, but extended over at least 8 cycles. In the case of pCPDT–Fc P1 the irreversible capacity remained higher than 10 mA h g^−1^ all throughout cycling. The potential profile of pCPDT–Fc P1 in Fig. 5a reveals a second potential plateau during charge at around 3.70 to 3.85 V *vs.* Li^+^/Li in addition to the regular plateau for the oxidation of ferrocene between 3.50 and 3.65 V *vs.* Li^+^/Li. This second plateau was very pronounced in the first cycle and then gradually lost in capacity. Since no counter-plateau was observed during discharge, it must be related with an irreversible process (in line with the irreversible capacity mentioned above). A similar irreversible plateau was observed for polymer pDTP–Fc P2 (Fig. 5b). Here, however, it decreased faster than for pCPDT–Fc P1, again in line with the irreversible capacity data.

This irreversible plateau at high potential most probably indicates over-oxidation of the pCPDT- or pDTP-backbone. Over-oxidation results in a disruption of the conjugation of the polymer chain and a decrease of the electrochemical activity. This behaviour has already been reported for polythiophene and for other conductive polymers before.^23^ With continuous cycling, the concentration of un-overoxidised polymer decreases and therefore further over-oxidation and irreversible capacity should decrease, which fits to the trend observed in the capacity curves. The major part of the reversible capacity stems from the Fc units and the reversible capacity should be only weakly affected by the degradation of the polyheteroacene backbone (as long as the Fc units remain electronically connected), which is in line with the good cycling stability. In principle, over-oxidation should decrease the electronic conductivity of the polymer-backbone and increase the electrode over-potentials. For the present electrodes (with 33 wt% carbon black as conductive additive) this does not seem to have a major effect, as no peak shifts are observed in the CVs (with peak currents exceeding a 1C rate) and the charge/discharge potential hysteresis does not increase during galvanostatic cycling (at a current of 0.1C). Obviously, the irreversible capacity related with over-oxidation could be avoided by lowering the end-of-charge potential. This would, however, also decrease the reversible capacity due charging of the Fc moiety, which proceeds up to 3.9 V *vs.* Li^+^/Li.

A further contribution to the irreversible capacity could stem from self-discharge, resulting in increased charging periods and decreased discharging periods. One well-known problem of organic electrode materials is their solubility in the electrolyte.^24^ Once dissolved, the molecules will act as redox shuttles between the electrodes and cause self-discharge (the redox shuttle molecule is oxidised at the cathode, diffuses to the anode, is reduced at the anode, diffused back to the cathode, and so on). The rate of self-discharge will depend on the concentration of dissolved molecules and on their diffusion coefficient in the electrolyte.^25^

## Conclusion

In this work, redox-active ferrocene moieties were covalently attached on 4*H*-cyclopenta-[2,1-*b*:3,4-*b*′]dithiophene (M1) or 4*H*-dithieno[3,2-*b*:2′,3′-*d*]pyrrole (M2), respectively. The electrochemical polymerization of monomers M1 and M2 led to very stable films of conducting polymers P1 and P2 exhibiting similar HOMO and LUMO energy levels. In the cyclic voltammograms of P1 and P2 a distinct reversible wave due to the ferrocene substituent was observed on top of a broad current response concomitant with the oxidation and reduction of the polymeric backbone. Oxidative chemical polymerization of M1 and M2 afforded the polymers P1 and P2 in larger amounts, which could hence be applied as cathode active materials in organic batteries. During galvanostatic cycling in a typical battery environment, ferrocene-modified polymers P1 and P2 showed reversible capacities of about 59.8 mA h g^−1^ meaning 90% and 87% of their theoretical capacity, respectively. The strategy of immobilizing ferrocene on an insoluble conjugated polymer in P1 and P2 largely decreased the self-discharging during the battery tests. Thus, excellent capacity retentions of 84% and 97% over 50 cycles were observed, which rank among the highest values reported for organic battery materials, so far.^5*c*^ The practical application of these polymers is hampered by the onset of over-oxidation of the polyheteroacene backbone at high states of charge, which results in low coulombic efficiencies. Limiting the end-of-charge potential to 3.7 V *vs.* Li+/Li would prevent this over-oxidation, but would also decrease the reversible capacity.

## Conflicts of interest

There are no conflicts to declare.

## Supplementary Material

RA-008-C8RA00129D-s001

RA-008-C8RA00129D-s002
