# Supplementary material for: Ferrocene-functionalized polyheteroacenes for the use as cathode active material in rechargeable batteries
Source: RSC Adv. 2018 Apr 17;8(26):14193–200. doi: 10.1039/c8ra00129d (PMC9079884; doi:10.1039/c8ra00129d)
Supplement: RA-008-C8RA00129D-s001 [file RA-008-C8RA00129D-s001.pdf]

Electronic Supplementary Material (ESI) for RSC Advances.
This journal is © The Royal Society of Chemistry 2018

Supplementary Information

Ferrocene-functionalized polyheteroacenes for the use as cathode active material in rechargeable batteries

Pierre-Olivier Schwartz,[a]* Sebastian Förtsch,[a] Elena Mena-Osteritz,[a] Dagmar Weirather-Köstner,[b] Mario Wachtler[b] and Peter Bäuerle[a]*

[a] Institute of Organic Chemistry II and Advanced Materials
Ulm University, Albert-Einstein-Allee 11, 89081 Ulm, Germany
[b] ZSW – Zentrum für Sonnenenergie- und Wasserstoff-Forschung Baden-Württemberg
Helmholtzstr. 8, 89081 Ulm, Germany
Fax (+49) 731-502-2840
Corresponding authors: pierreolivier.schwartz@gmail.com; peter.baeuerle@uni-ulm.de

Experimental methods

Instrument and measurement

NMR spectra were recorded on a Bruker Avance 400 (^{1}H NMR: 400 MHz, ^{13}C NMR: 125 MHz) at 25 °C. Chemical shift values (δ) are expressed in parts per million using residual solvent protons (^{1}H NMR, δ_H = 7.26 for $CDCl_3$; δ_H = 3.31 for CD_3OD; ^{13}C NMR: δ_C = 77.16 for $CDCl_3$; δ_C = 49.00 for CD_3OD) as internal standard. The splitting patterns are designated as follows: s (singlet), d (doublet), t (triplet), m (multiplet). Coupling constants (*J*) relate to proton-proton couplings. GC/EI-MS (70 eV) measurements were performed on a Shimadzu GC-MS-QP2010 SE. Chemical ionisation (CI) mass spectra were measured on a Finnigan MAT SSQ-7000. Melting points were determined using a Büchi B-565 and a Mettler Toledo DSC 823^e. Thermogravimetric analyses (TGA) were carried out with a TGA/SDTA 851^e from Mettler Toledo. Elemental analyses were performed on an Elementar Vario EL. FT-IR spectra were measured with a Perkim Elmer Spectrum 2000.

The diffraction data of a brownish crystal of **M1** were collected in a stream of nitrogen at 180 K on a Agilent SuperNova, Cu at zero, Atlas CCD using graphite-monochromated Cu Kα radiation. Data collection strategy was performed with the APEX2 software, data reduction,

absorption correction and cell refinement with CrysAlisPro171. The structure was solved by direct methods with SHELXL-2016/6, revealing all atoms of **M1**. H atoms were discernible from difference Fourier maps during refinement on F2 with SHELXL-97. For the final model, all atoms were refined anisotropically.

Cyclic voltammetry experiments were performed with a computer-controlled Autoloab PGSTAT30 potentiostat in a three-electrode single-compartment cell with a platinum working electrode, a platinum wire counter electrode, and a Ag/AgCl reference electrode. All potentials were internally referenced to the ferricenium/ferrocene (Fc^+/Fc) redox couple. Spectroelectrochemical measurements of the polymer films were carried out in a 0.1 M solution of tetrabutylammonium hexafluorophosphate in dry acetonitrile. The applied setup has been described in the literature.[1] A platinum working electrode, a Ag/AgCl reference electrode, and a platinum sheet as the counter electrode were used. During the recording of the UV-Vis-NIR spectra the applied potential was kept constant. Instrumental artefacts were removed and marked in the spectra. Moreover, the absorption in the UV-Vis-NIR region below 860 nm was adjusted to the absorption at higher wavelengths to obtain continuous spectra. The UV-Vis-NIR spectra were recorded on a Perkin Elmer Lambda 19 spectrometer.

Materials

Copper(II) tetrafluoroborate was provided from *Aldrich*. *N,N′*-dicyclohexylcarbodiimide (DCC), 4-dimethylaminopyridine (DMAP) and ferrocenecarboxylic acid **3** were provided from *Alfa Aesar*. Solvents were purchased from *Merck* and *Aldrich,* purified, and dried by standard methods prior to use. 4*H*-cyclopenta[2,1-*b*:3,4-*b*′]dithiophen-4-one[2] and 4*H*-dithieno[3,2-*b*:2′,3′-*d*]pyrrole 2[3] were prepared according to literature procedures.

Syntheses

4*H*-cyclopenta[2,1-*b*:3,4-*b′*]dithiophen-4-ol (1). 4*H*-cyclopenta[2,1-*b*:3,4-*b′*]dithiophene-4-one (230 mg, 1.2 mmol), dry THF (7 mL) and anhydrous $NiCl_2$ (0.5 g, 3.6 mmol) were placed in a Schlenk tube under argon. $NaBH_4$ (0.4 g, 10.8 mmol) was added and the formation of a black precipitate of nickel boride was observed immediately. The reaction mixture was stirred at room temperature and its progress was monitored by TLC for disappearance of starting material. The reaction mixture was diluted with dichloromethane and filtered through a celite pad. The filtrate was diluted with water and extracted with CH_2Cl_2 (3×). The

combined organic layers were dried over Na_2SO_4, concentrated, and purified by column chromatography (*n*-hexane:EtOAc 1:9). The desired compound was obtained as a white solid (210 mg, 1.08 mmol, 90%). Mp 165.7-167.1°C. ^{1}H NMR (CD_3OD, ppm): δ = 7.25 (d, 2H, *J* = 4.9 Hz), 7.13 (d, 2H, *J* = 4.9 Hz), 5.26 (s, 1H). ^{13}C NMR (CD_3OD, ppm): δ = 154.7, 139.1, 126.5, 123.9, 70.4. FT-IR (KBr, 25 °C, cm^{-1}): ν = 3370, 3303, 3076, 1322, 1299, 1241, 1210, 1104, 1050, 921, 801, 712, 690. Elemental analysis: calc. (%) for $C_9H_6OS_2$: C 55.64, H 3.11, S 33.01; found: C 56.03, H 2.95, S: 32.19.

4-Ferrocene-4*H*-cyclopenta[2,1-*b*:3,4-*b′*]dithiophene (CPDT-Fc, M1). In a closed Schlenk tube 4*H*-cyclopenta[2,1-*b*:3,4-*b′*]dithiophene-4-ol **1** (140 mg, 0.7 mmol) and ferrocene carboxylic acid **3** (232 mg, 1 mmol) were dissolved in dry CH_2Cl_2 (15 mL). The solution was stirred at room temperature and a mixture of *N,N′*-dicyclohexylcarbodiimide (205 mg, 1 mmol) and 4-dimethylaminopyridine (150 mg, 0.8 mmol) were added in one portion. The solution was stirred at room temperature for two days, filtrated through a pad of celite, and concentrated. The crude product was purified by column chromatography (petroleum ether:EtOAc 5:1), followed by recrystallization from *n*-hexane. The desired monomer **M1** was provided as orange needles (270 mg, 0.66 mmol, 92%). Mp 140.4°C (DSC). ^{1}H NMR ($CDCl_3$, ppm): δ = 7.20 (d, *J* = 4.9 Hz, 2H), 7.16 (d, *J* = 4.9 Hz, 2H), 6.38 (s, 1H), 4.85 (t, *J* = 1.9 Hz, 2H), 4.42 (t, *J* = 1.9 Hz, 2H), 4.19 (s, 5H). ^{13}C NMR ($CDCl_3$, ppm): δ = 127.3, 149.2, 139.7, 125.6, 123.7, 71.6, 70.4, 69.9, 69.5. FT-IR (KBr, 25 °C, cm^{-1}): ν = 3096, 2920, 1707, 1653, 1636, 1561, 1459, 1370, 1265, 1134, 1039, 933, 819, 702, 699, 503, 498, 479, 432, 412. Elemental analysis: calc. (%) for $C_{20}H_{14}FeO_2S_2$: C 59.10, H 3.47, S 15.78; found: C 59.09, H 3.52, S 15.21. GC/EI-MS: *m/z* = 406 $[M + H]^+$, 177 $[M–OCOFc + H]^+$.

Poly(4-ferrocene-4*H*-cyclopenta[2,1-*b*:3,4-*b′*]dithiophene) (pCPDT-Fc, P1). A mixture of CPDT-Fc **M1** (250 mg, 0.6 mmol) and $Cu(BF_4)_2$ (583 mg, 2.2 mmol) in acetonitrile (20 mL) was heated at 60 °C for 2 days. At the end of the reaction, a black precipitate was obtained, isolated via centrifugation, and dried in vacuo. Then, the black polymer was successively washed with acetonitrile, water, methanol, and chloroform. The desired pCPDT-Fc **P1** was isolated as a black powder (180 mg, 0.44 mmol, 73%). FT-IR (KBr, 25 °C, cm^{-1}): ν = 3085, 2925, 1715, 1454, 1374, 1264, 1128, 1056, 946, 916, 820, 764, 504, 499, 483, 468.

4-Ferrocene-4*H*-dithieno[3,2-*b*:2′,3′-*d*]pyrrole (DTP-Fc, M2). A suspension of the unsubstituted DTP **2** (430 mg, 2.4 mmol), ferrocene carboxylic acid **3** (732 mg, 3.1 mmol), *N,N′*-dicyclohexylcarbodiimide (644 mg, 3.1 mmol), and 4-dimethylaminopyridine (323 mg,

2.6 mmol) in dichloromethane (20 mL) was stirred at room temperature for two days. The reaction mixture was filtrated and concentrated under reduced pressure. The obtained residue was purified by column chromatography on silica gel (petroleum ether:CH_2Cl_2 3:1) to afford the desired monomer **M2** as an orange solid (907 mg, 2.32 mmol, 97%). Mp 122-124 °C. ^{1}H NMR ($CDCl_3$, ppm): δ = 7.21-7.15 (m, 4H), 4.95-4.93 (m, 2H), 4.55-4.54 (m, 2H), 4.28 (s, 4H). ^{13}C NMR ($CDCl_3$, ppm): δ = 168.9, 143.4, 124.1, 120.7, 116.8, 76.6, 72.4, 71.2, 71.1. FT-IR (KBr, 25 °C, cm^{-1}): ν = 2976, 2934, 2837, 1727, 1614, 1556, 1461, 1372, 1333, 1291, 1255, 1233, 1209, 1176, 1107, 1048, 1027, 856, 836, 779, 754, 671, 581, 567, 503. Elemental analysis: calc. (%) for $C_{19}H_{13}FeNOS_2$: C 58.32, H 3.35, N 3.58, S 16.39; found: C 58.12, H 3.53, N 3.81, S 16.32. CI-MS: *m/z* (%) = 391 (81) $[M + H]^+$.

Poly(4-ferrocene-4*H*-dithieno[3,2-*b*:2',3'-*d*]pyrrole) (pDTP-Fc, P2). Copper(II) tetrafluoroborate (1.61 g, 6.8 mmol) was added to a solution of DTP-Fc **M2** (665 mg, 1.7 mmol) in dichloromethane (15 mL) and acetonitrile (15 mL). The reaction mixture was heated at 50 °C for two days. Afterwards, the black suspension was filtrated and the filter residue was washed with several solvents (methanol, water, petroleum ether, THF, CH_2Cl_2, and ether) to afford the desired polymer **P2** as a dark blue solid (442 mg, 1.14 mmol, 67%). FT-IR (KBr, 25 °C, cm^{-1}): ν = 2922, 1637, 1444, 1375, 1296, 1106, 815, 745, 654, 488, 424, 406.

X-Ray single crystal structure analysis of CPDT-Fc (M1)

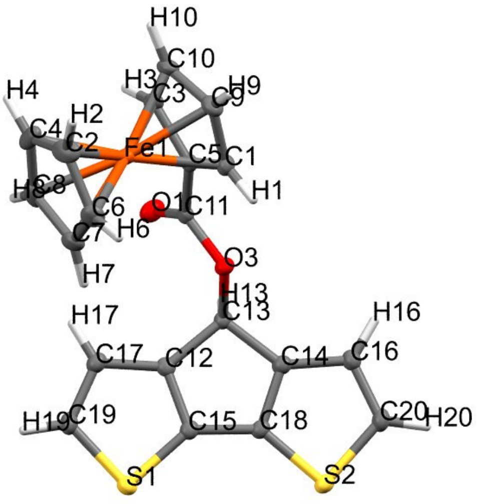

Figure S1: Crystal structure of **M1** with atomic labels**.** Displacement ellipsoids are drawn at the 50% probability level.

Table S1: X-ray crystallographic data for **M1**

Empirical formula	$C_{20}H_{14}FeO_2S_2$
Formula weight	406.28
Temperature/K	149.95(10)
Crystal system	orthorhombic
Space group	$P2_12_12_1$
a/Å	5.86404(10)
b/Å	10.14209(16)
c/Å	27.2116(4)
α, β, γ/°	90
Volume/Å^3	1618.37(4)
Z	4
ρ_{calc}g/cm^3	1.667
μ/mm^{-1}	1.201
F(000)	832.0
Crystal size/mm^3	0.2371 × 0.2102 × 0.1142
Radiation	MoKα (λ = 0.71073)
2Θ range for data collection/°	5.988 to 58.976
Index ranges	-7 ≤ h ≤ 7, -12 ≤ k ≤ 13, -34 ≤ l ≤ 34
Reflections collected	15457
Independent reflections	3954 [R_{int} = 0.0332, R_{sigma} = 0.0330]
Data/restraints/parameters	3954/0/283
Goodness-of-fit on F^2	1.083
Final R indexes [I>=2σ (I)]	R_1 = 0.0255, wR_2 = 0.0513
Final R indexes [all data]	R_1 = 0.0287, wR_2 = 0.0528

Table S2: Selected bond distances, angles, and torsion angles from the crystal structure analysis of **M1**. Atom labels correspond to those on Figure S1.

Atom1	Atom2	Distance (Å)
C19	C17	1.367(3)
C17	C12	1.410(4)
C12	C15	1.372(3)
C19	S1	1.724(3)
S1	C15	1.709(2)
C15	C18	1.447(3)
C12	C13	1.518(3)
C18	C14	1.380(3)
C14	C13	1.511(3)
C14	C16	1.415(3)
C16	C20	1.359(4)
C20	S2	1.730(3)
S2	C18	1.705(2)
C13	O3	1.454(3)
O3	C11	1.348(3)
C11	O1	1.212(3)
C11	C5	1.472(3)
C5	C1	1.430(4)
C1	C9	1.417(4)
C9	C10	1.423(4)
C10	C3	1.416(4)
C3	C5	1.430(3)
C5	Fe1	2.034(2)
Fe1	C4	2.033(3)
C4	C8	1.415(4)
C8	C7	1.414(4)
C7	C6	1.414(4)
C6	C2	1.420(4)

Atom1	Atom2	Atom3	Atom4	Torsion (°)
S1	C15	C18	S2	2.8(4)
C17	C12	C13	O3	57.7(4)
C16	C14	C13	O3	-58.9(3)
C12	C13	O3	C11	-83.7(2)
C13	O3	C11	O1	-1.4(3)
C13	O3	C11	C5	180.0(2)
O3	C11	C5	C1	-3.7(4)

Atom1	Atom2	Atom3	Angle (°)
C19	C17	C12	111.2(2)
C17	C12	C15	113.4(2)
C12	C15	S1	111.7(2)
C15	S1	C19	91.0(1)
S1	C19	C17	112.7(2)
C17	C12	C13	137.1(2)
C12	C13	C14	102.0(2)
C13	C14	C18	109.7(2)
C14	C18	C15	108.9(2)
C18	C15	C12	109.5(2)
C13	C14	C16	137.9(2)
C14	C16	C20	111.9(2)
C16	C20	S2	112.7(2)
C20	S2	C18	90.7(1)
S2	C18	C14	112.4(2)
S1	C15	C18	138.7(2)
C15	C18	S2	138.7(2)
C12	C13	O3	112.8(2)
C13	O3	C11	114.9(2)
C14	C13	O3	111.3(2)
O3	C11	O1	124.0(2)
O3	C11	C5	111.8(2)
C11	C5	C1	126.9(2)
C5	C1	C9	107.3(2)
C1	C9	C10	108.5(2)
C9	C10	C3	108.4(2)
C10	C3	C5	107.4(2)
C3	C5	C1	108.4(2)
C5	Fe1	C7	112.7(1)
C7	C8	C4	107.7(2)
C8	C4	C2	108.3(3)
C4	C2	C6	108.2(3)
C2	C6	C7	107.5(2)
C6	C7	C8	108.4(2)

Table S3: Intermolecular short contact lengths (below van der Waals distances). Atom labels correspond to those on Figure S1.

Atom in molecule 1	Atom in molecule 2	Length (Å)	Symmetry operation1	Symmetry operation2
S2	C19	3,486	x,y,z	-1+x,y,z
C20	C3	3,349	x,y,z	-1+x,y,z
H5	H18	2,386	x,y,z	-1+x,y,z
S1	H19	2,98	x,y,z	-1/2+x,1.5-y,1-z
C12	H19	2,891	x,y,z	-1/2+x,1.5-y,1-z
C19	H19	2,804	x,y,z	-1/2+x,1.5-y,1-z
H4	C8	2,843	x,y,z	-x,1/2+y,1/2-z

Infrared spectra of CPDT-Fc (M1), pCPDT-Fc (P1), DTP-Fc (M2), and pDTP-Fc (P2)

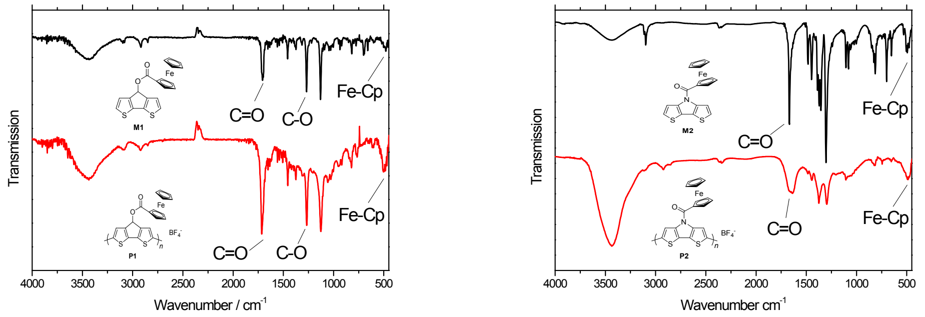

Figure S2: Infrared spectra (KBr pellets, 25 °C) of CPDT-Fc **M1** (left, black) and pCPDT-Fc **P1** (left, red), respectively DTP-Fc **M2** (right, black) and pDTP-Fc **P2** (right, red).

TGA and DSC measurements of pCPDT-Fc (P1) and pDTP-Fc (P2)

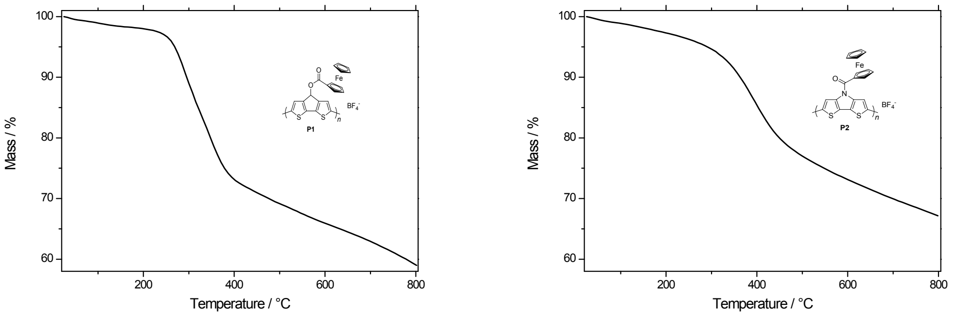

Figure S3: TGA measurements of pCPDT-Fc **P1** (left) and pDTP-Fc **P2** (right).

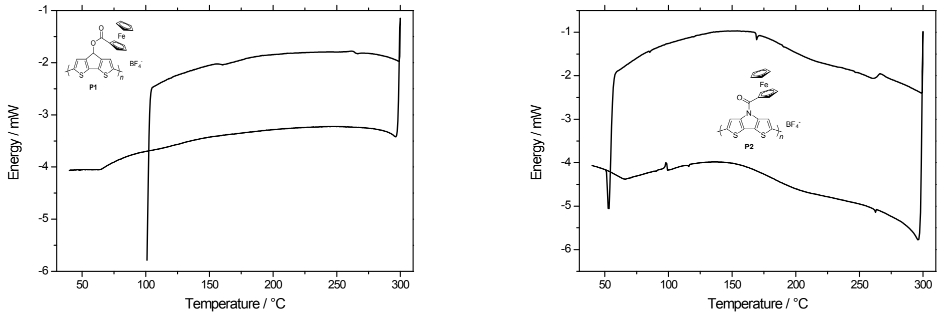

Figure S4: DSC measurements of pCPDT-Fc **P1** (left) and pDTP-Fc **P2** (right).

Cyclic voltammetry of monomers M1 and M2

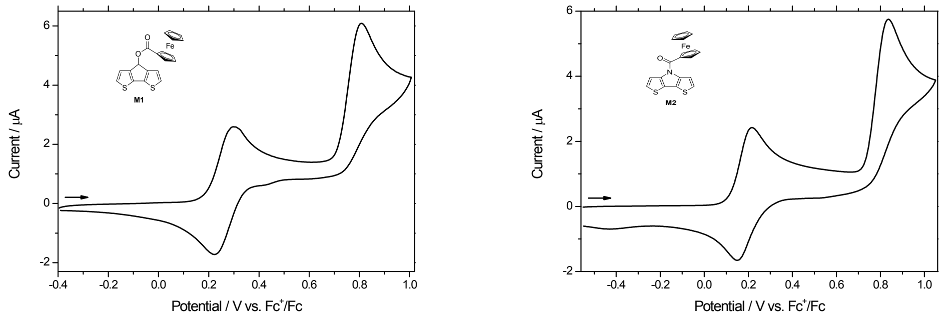

Figure S5: Cyclic voltammograms of CPDT-Fc **M1** (left) and DTP-Fc **M2** (right) with a scan rate of 100 mV/s in 0.1 M $TBAPF_6$ in acetonitrile.

Cyclic voltammetry of polymers P1 and P2

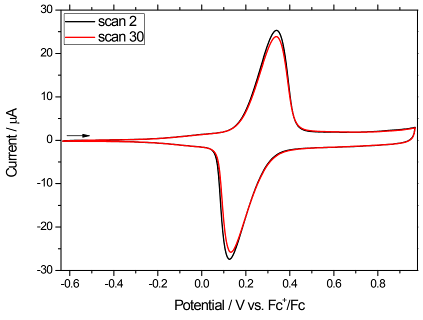

Figure S6: Cyclic voltammograms of pCPDT-Fc **P1** in 0.1 M $TBAPF_6$ in CH_2Cl_2 conducting 30 scans with a scan rate of 100 mV/s.

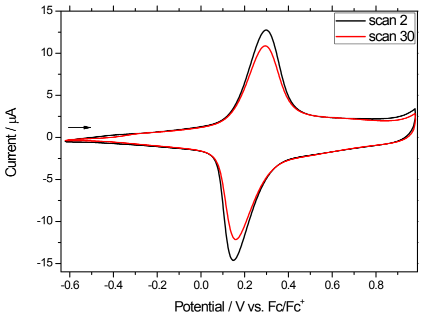

Figure S7: Cyclic voltammograms of pDTP-Fc **P2** in 0.1 M $TBAPF_6$ in CH_2Cl_2 conducting 30 scans with a scan rate of 100 mV/s.

Electrode preparation and battery tests

Composite electrodes with a composition of 56.6 wt.% active material (PCPDT-Fc or PDTP-Fc), 33.0 % carbon black, and 10.4 wt.% poly(tetrafluoroethylene) (PTFE) were prepared as follows: Approx. 100 mg active material and 60 mg carbon black (Imerys, Super P Li) were pre-mixed by grinding in an agate mortar. Then, the appropriate amount of PTFE powder (Dyneon, TF 2025Z) was added under further grinding, to obtain a plastic mass, which was then calendered into a free-standing film by roll pressing. Electrodes with 12 mm diameter and mass loadings between 6.5 and 7.5 g/cm^2 were punched out and pressed onto Al expanded metal grid as current collector, and finally dried at 80 °C under dynamic vacuum (~ 1 mbar) overnight.

T-type 3-electrode cells were built from these electrodes and Li metal as counter and reference electrodes using 1 M $LiPF_6$ / ethylene carbonate (EC): dimethyl carbonate (DMC) (1:1 by wt.) (Ube Industries) as electrolyte and 3 layers of borosilicate glass microfibre non-wovens (Whatman, GF/A) as separator.

Cyclic voltammograms were recorded with a VMP3 potentiostat from Biologic, using a scan rate of 0.1 mV/s. Galvanostatic cycling tests were performed with a CTS-Lab cycler from Basytec, using a current rate of 0.1C and cut-off potentials of 2.5 / 2.7 and 3.9 V vs. Li^+/Li. (A current rate of 1C corresponds to the current which is needed to charge or discharge the theoretical capacity of the material in 1 h. For instance, the theoretical capacity of PCPDT-Fc is 66.3 mAh/g, therefore the 1C rate is 66.3 mA/g, and the 0.1C rate is 6.63 mA/g.) The specific capacities mentioned in the text refer to the mass of active material only.

References

[1] J. Salbeck, *J. Electroanal. Chem.,* 1992, **340**, 169-195.

[2] S.-Y.Liou, C.-S. Ke, J.-H. Chen, Y.-W. Luo, S.-Y. Kuo, Y.-H. Chen, C.-C. Fang, C.-Y. Wu, C.-M. Chiang, Y.-H. Chan, *ACS Macro Letters,* 2016, **5**, 154-157.

[3] S. Förtsch, A. Vogt, P. Bäuerle*, J. Phys. Org. Chem*, *2017*, **30**, e3743.
